# 

**Published:** 1930

**Authors:** 


### The Medical Library of the University of Bristol,

WITH WHICH IS INCORPORATED

**The Library of the Bristol Medico-Chirurgical Society.**

---

*The following donations have been received since the publication of the list in June,* 1930.

October, 1930.

| | |
|---|---|
| American Laryngological, Rhinological and Otological Society (1) .. .. .. | 1 volume |
| American Otological Society (2) .. .. | 1 ,, |
| Association des Médecins de Langue Française (3) .. .. .. .. .. .. | 1 ,, |
| Dr. W. S. Bainbridge (4) .. .. .. | 1 ,, |
| British Medical Association (5) .. .. | 1 pamphlet |
| Director-General, Army Medical Services (6) | 1 volume |
| Professor E. W. Hey Groves (7) .. .. | 11 volumes |
| Japanese Embassy (8) .. .. .. .. | 2 ,, |
| Munro Smith Memorial Fund (9) .. .. | 1 volume |
| Philadelphia Committee for Clinical Study of Opium Addiction (10) .. .. .. | 1 ,, |
| Rockefeller Foundation (11) .. .. .. | 1 ,, |
| Surgeon-General of U.S. Army (12) .. .. | 1 ,, |
| University of Glasgow (13) .. .. .. | 1 ,, |
| University of Otago (14) .. .. .. | 1 ,, |

Unbound periodicals have also been received from Professor Hey Groves.

### THE ONE HUNDRED AND THIRTY-THIRD LIST OF BOOKS.

The figures in round brackets refer to the figures after the names of the donors. The books to which no such figures are attached have either been bought from the Library Fund or received through the *Journal.*

| | | | |
|---|---|---|---|
| **Adachi, B.** .. .. .. | *Das Arteriensystem der Japaner.* 2 Vols. | (8) | 1928 |
| **Ballance, C.** .. .. .. | *Dawn and Epic of Neurology and Surgery* | (13) | 1930 |
| **Bland-Sutton, Sir J.** .. | *Story of a Surgeon* .. .. .. .. .. .. | (7) | 1930 |
| **Beeston, G. N.** .. .. | *Conjoint Finals* .. .. .. .. .. .. .. .. .. | | 1930 |
| **Bourne, A.** .. .. .. | *Recent Advances in Obstetrics and Gynæcology* | (9) | 1928 |
| *B.M.A. Proposals for a General Medical Service for the Nation* .. | | (5) | 1930 |
| **Brockbank, E. M.**.. .. | *Diagnosis and Treatment of Heart Disease* 6th Ed. | | 1930 |

**Bruns, O., and Thiel, K.** *Die Wiederbelebung* .. .. .. .. .. .. (7) 1930
**Gibson, A. G.** .. .. *Mycoses of the Spleen* .. .. .. .. .. .. .. 1930
**Gordon-Taylor, G.** .. *The Dramatic in Surgery* .. .. .. .. .. .. 1930
**Gordon, R. G., and Thomson, F. G.** *Physiological Principles of Hydrology* 1930
**Haumann, W.** .. .. *Die Wirbelbruche und ihre Endergebnisse*.. (7) 1930
**Hermannsdorfer, A.** .. *Die Diätetische Vor-und Nachkur bei der Operativen Behandlung der Lungentuberkulose* (7) 1929
**Groves, E. W. H.** .. *Synopsis of Surgery* .. .. .. .. .. 9th Ed. 1930
**Kromayer, E.** .. .. *Cosmetic Treatment of Skin Complaints* .. .. 1930
**Larkin, A. J.** .. .. *Radium in General Practice* .. .. .. .. .. 1929
**Leriche, A., and Policard, A.** *Physiologie, Pathologique Chirurgicale* (7) 1930
**Lloyd-Williams, A.** .. *The Doctor's Job* .. .. .. .. .. .. .. .. .. 1930
**Love, R. J. M.** .. .. *A Shorter Surgery* .. .. .. 2nd Ed. 1930
**McCann, F. J.** .. .. *Contraception a Common Cause of Disease* .. 1928
**Mackenzie, Sir C.** .. *Action of Muscles* .. .. .. .. .. 2nd Ed. 1930
**Martindale and Westcott** *Extra Pharmacopœia* Vol. 2. .. 19th Ed. 1929
*Modern Technique in Treatment.* Vols. 1 and 2 .. .. .. .. .. .. 1928
**Moncrieff, A.** (ed.) .. *Nursing and Diseases of Children* .. .. .. 1930
**Nauth, B.** .. .. .. *All-India Medical Conference* .. .. .. (7) 1929
*Opium Addiction* .. .. .. .. .. .. .. .. .. .. .. .. .. .. .. (10) 1929
**Pellegrini, A.** .. .. *Cinematizzioni* .. .. .. .. .. .. .. .. (7) 1929
*Pharmacopœia of the Central London Ophthalmic Hospital*.. .. .. .. 1930
**Ray, K. S.** .. .. .. *Some Problems of Medical Profession in India* (7) —
**Sauerbruch, F.** .. .. *Chirurgie der Brustorgane.* I.i. .. .. (7) 1930
**Scott, G. L.** .. .. .. *The Morphine Habit* .. .. .. .. .. .. .. 1930
**Sechehaye, A.** .. .. *The Treatment of Tuberculosis with Umckaloabo* —
**Smith, F.** .. .. .. *Short History of the R.A.M.C.* .. .. .. (6) 1929
**Stuart-Low, G.** .. .. *Nasal Catarrh* .. .. .. .. .. .. .. .. .. 1930

### TRANSACTIONS, REPORTS, JOURNALS, ETC.

Association des Médecins de Langue Française Congrés, 1929 .. .. —
Collected Papers of the Lockwood Clinic, 1929 .. .. .. .. .. .. (7) —
Collected Papers of the Mayo Clinic. Vol. 21 .. .. .. .. .. .. .. 1929
Index Catalogue Surgeon-General's Library. 3rd Series, Vol. 8 .. (12) 1929
Medical Annual, 1930 .. .. .. .. .. .. .. .. .. .. .. .. .. .. —
Methods and Problems of Medical Education. 17th Series .. .. (11) 1930
Proceedings of the University of Otago Medical School. Vol. 7 .. (14) 1930
Progressive Medicine. June, 1930 .. .. .. .. .. .. .. .. .. .. .. —
Report of 5th International Congress of Military Medicine and Pharmacy, 1929 .. .. .. .. .. .. .. .. .. .. .. .. .. .. .. .. .. .. (4) —
St. Thomas's Hospital Reports. Vol. 52 .. .. .. .. .. .. .. .. .. 1930
*Société Internationale de Chirurgie.* VIIIe Congres. Vol. 1 .. .. (7) 1929
Surgical Clinics of N. America. Vol. 10. Nos. 3 and 4 .. .. .. .. .. 1930
Transactions of the American Laryngological, Rhinological and Otological Society. Vol. 35 .. .. .. .. .. .. .. .. .. .. .. .. .. (1) 1929
Transactions of the American Otological Society. Vol. 19 .. .. (2) 1929
Transactions of the College of Physicians of Philadelphia. 3rd Series, Vol. 51 1929

### Local Medical Notes.

**Long Fox Memorial Lecture.**—The Vice-Chancellor, Thomas Loveday, LL.D., in the Chair. The Eighteenth Annual Lecture will be delivered on Tuesday, October 28th, 1930, at 5.15 p.m., in the Physiological Lecture Theatre of the University, by Professor J. A. Nixon, C.M.G., M.D., F.R.C.P. Subject: "The Influence of Food on the Production and Prevention of Disease." Tea will be served at 4.30 p.m. for those intimating to the Dean of the Medical Faculty their intention of being present.

EXAMINATION RESULTS.

**University of Bristol.**—Students of the University have recently passed the following examinations:—

M.B., Ch.B.—*First Examination. Pass:* F. C. Collingwood, Theodora M. Crabtree, A. E. Jowett, S. D. Loxton, M. A. Nicholson, B. Ridgway. *In Physics (Completing Examination):* J. R. G. Damrel, Irene G. Hamilton, T. H. Taylor, Ursula W. Wood. *In Biology (Completing Examination):* A. C. Molden. *In Botany (Completing Examination):* A. J. Matheson.

B.D.S.—*First Examination. Pass:* Kathleen G. Brimelow.

L.D.S.—*Preliminary Science Examination. Pass: In Biology (Completing Examination):* J. G. Clancy, R. J. Lee.

Conjoint Board.—L.D.S., R.C.S.—*Final Examination. Part I. Pass:* I. M. Ayliffe, D. R. Jones. *Part II. Pass:* W. Forsyth, W. F. G. Harvey, W. R. R. Mewton, K. V. M. Taylor-Milton.

### Publications Received.

From Erik Agduhr:
*The Appearance of the Electrocardiogram in Heart Lesions produced by Cod Liver Oil Treatment* (from *Acta Pædiatrica*, Vol. 8, 9, 10). By Erik Agduhr and Nils Stenstrom.

From John Bale, Sons and Danielsson Ltd.
*General Practice (Some Further Experiences)*. By Ernest Ward, M.D., F.R.C.S.
*Contraception a Common Cause of Disease*. By F. J. McCann, M.D., F.R.C.S. 1928. Price 1s. net.

From Cassell & Co. Ltd.:
*Sick Children: Diagnosis and Treatment. A Manual for Students and Practitioners*. By Donald Paterson, M.D. (Edin.), F.R.C.P. (Lond.).

From The Director-General, Army Medical Services:
*A Short History of the Army Medical Corps*. By Colonel F. Smith, C.B., C.M.G., D.S.O. 1929.

From William Heinemann Ltd.:
*After Consulting Hours*. By Christopher Howard, M.R.C.S. (Eng.), L.R.C.P. (Lond.).
*Shorter Convalescence*. By Lieut.-Col. J. K. McConnel, D.S.O., M.C. 1930. Price 5s. net.
*On Faith and Science of Surgery*. By Sir John Bland-Sutton, Bart. 1930. Price 7s. 6d. net.

From "The Lancet" Ltd.:
*Guy's Hospital Reports*. Vol. 80 (Vol. 10, fourth series), No. 3. July, 1930. Edited by Arthur F. Hurst, M.D.
*Guy's Hospital Reports*. April, 1930. Annual subscription, £2 2s. 12s. 6d. net per issue.

From H. K. Lewis & Co. Ltd.:
*Excessive Menstrual Bleeding, its Treatment by X-rays. Management of Patients suffering from Cancer of the Breast*. By Francis Hernaman-Johnson, M.D., D.M.R.E.
*Essays and Addresses, Sociological, Biological and Psychological*. By A Surgeon. 1930. Price 10s. 6d. net.
*The Theory and Practice of Nursing*. By M. A. Gullan. 3rd Edition. 1930. Price 9s. net.
*The Elements of Medical High-Frequency and Diathermy. For Assistants and Nurses*. By W. Claughton Douglass, M.R.C.S., D.M.R.E. 1930. Price 6s. net.

From Lister Medical Press:
*The Treatment of Skin Diseases in Detail*. By Noxon Toomey, M.D., B.A., F.A.C.P.

From E. & S. Livingstone:
*Handbook of Therapeutics*. By David Campbell, M.C., M.A., B.Sc., M.D. 1930. Price 12s. 6d. net.

From Humphrey Milford:
*Aphasia in Children*. By Alex. W. G. Ewing, M.A., Ph.D.
*Histology for Medical Students*. By H. Hartridge and F. Haynes.

From Kegan Paul, Trench, Trubner & Co. Ltd.:
*Cancer of the Larynx*. By Sir St. Clair Thomson, M.D., F.R.S.C., F.R.C.P. and Lionel Colledge, M.B., F.R.S.C.
*Individual Diagnosis*. By F. G. Crookshank, M.D., F.R.C.P.

From John Wright & Sons Ltd.:
*Cancer of the Lung and other Intrathoracic Tumours*. By Maurice Davidson, M.A., M.D., B.Ch., F.R.C.P.
*Chronic Nasal Sinusitis and its Relation to General Medicine*. By P. Watson-Williams, M.D. 1930. Price 15s. net.
*British Journal of Surgery*. July, 1930.
*Congenital Club-Foot (Talipes Equinovarus)*. By E. P. Brockman, M.Ch., F.R.C.S. 1930. Price 10s. 6d. net.

